# Treatment patterns and survival outcomes in elderly patients with intrahepatic cholangiocarcinoma: NCDB analysis

**DOI:** 10.3389/fonc.2026.1857928

**Published:** 2026-07-20

**Authors:** Haytham Effarah, Jino Park, Garrett Harada, David Hong, Caressa Hui

**Affiliations:** 1 University of California Irvine School of Medicine, Irvine, CA, United States; 2 Department of Radiation Oncology, University of California Irvine Medical Center, Orange, CA, United States

**Keywords:** age, cholangiocarcinoma, elderly, intrahepatic, radiation, treatment escalation, trimodality

## Abstract

Intrahepatic cholangiocarcinoma (ICC) is a rare malignancy with a poor prognosis and rising incidence that is most commonly diagnosed in older adults. However, elderly patients remain underrepresented in clinical trials, limiting evidence to guide treatment selection in this population. Using the National Cancer Database, we evaluated age-associated differences in treatment patterns and overall survival in patients with nonmetastatic ICC diagnosed between 2004 and 2020, comparing elderly (>65 years) and younger (≤65 years) cohorts. Among 10,342 patients, 5,259 (50.9%) were elderly. Treatment groups included surgery only, chemotherapy only, radiotherapy only, chemotherapy plus radiotherapy, surgery plus chemotherapy or radiotherapy, and trimodality therapy. Among patients with operable disease, elderly patients were less likely to receive trimodality therapy (8.8% vs 14.4%) or surgery plus chemotherapy/radiotherapy (33.2% vs 41.5%) and more likely to undergo surgery alone (58.0% vs 44.1%). Among inoperable patients, elderly patients were less likely to receive chemotherapy-based treatment and more likely to receive radiotherapy alone (7.2% vs 2.8%). In both age groups, treatment strategies incorporating surgery were associated with significantly improved survival compared with nonsurgical approaches. In elderly patients, survival did not significantly differ among the 3 surgical treatment groups at 1, 2, or 5 years. Among nonsurgical elderly patients, chemotherapy plus radiotherapy was associated with improved 1-year (67.6% vs 58.3%) and 2-year overall survival (36.4% vs 26.0%) compared with chemotherapy alone, while radiotherapy alone produced outcomes comparable to chemoradiation. In summary, within the inherent limitations of a retrospective NCDB analysis, we observed that elderly patients with nonmetastatic ICC were more likely to receive treatment de-intensification in both surgical and nonsurgical settings. Furthermore, while surgery remained strongly associated with survival across all patients, in the inoperable setting, we observed that the addition of radiation therapy to the primary site was associated with improved short-term survival outcomes compared to those who only received systemic therapy. These observations should be considered hypothesis-generating and warrant further evaluation using more granular clinical, pathologic, and treatment-specific data.

## Introduction

Cholangiocarcinoma is a rare malignancy of the biliary tract system that is most commonly diagnosed between the sixth and eighth decades of life, with patients frequently presenting with advanced, unresectable disease (1). Intrahepatic cholangiocarcinoma (ICC), a distinct subset of these tumors, is associated with poor prognosis and is rising in incidence, increasing from 0.80 to 1.99 per 100,000 person-years between 2001 and 2017 (2). Standard management paradigms for ICC include surgical resection and adjuvant chemotherapy for operable disease and systemic therapy for unresectable cases (3, 4).

Despite ICC affecting older patient populations, elderly patients remain underrepresented in clinical trials, potentially diminishing their treatment options and limiting access to treatments that could confer survival benefit. Extrapolation of treatment regimens derived from younger, healthier populations who have significantly less comorbidities may also compromise patient safety for elderly patients.

Herein, we investigate historical treatment trends for ICC stratified by age at diagnosis, including the utilization of surgical resection, chemotherapy, and radiation therapy. The goals of this investigation are to identify age-associated differences in treatment patterns in patients with ICC, and to determine the optimal treatment strategy for elderly patients with ICC.

## Materials and methods

### Cohort selection

The National Cancer Database (NCDB) was established in 1989 as a joint program of the Commission on Cancer (CoC) American College of Surgeons and the American Cancer Society. The NCDB is a nationwide oncology database that captures approximately 70% of all newly diagnosed cancers in the United States annually from > 1,500 CoC accredited facilities and contains > 34 million historical records. Data elements are collected and submitted to the NCDB from commission-accredited oncology registries using standardized coding and data item definitions, including details not available from the Surveillance, Epidemiology, and End Results (SEER) registry, such as radiation dose and technique, and use of chemotherapy. This project was exempted by the institutional review board.

### Study population

**Figure 1:**
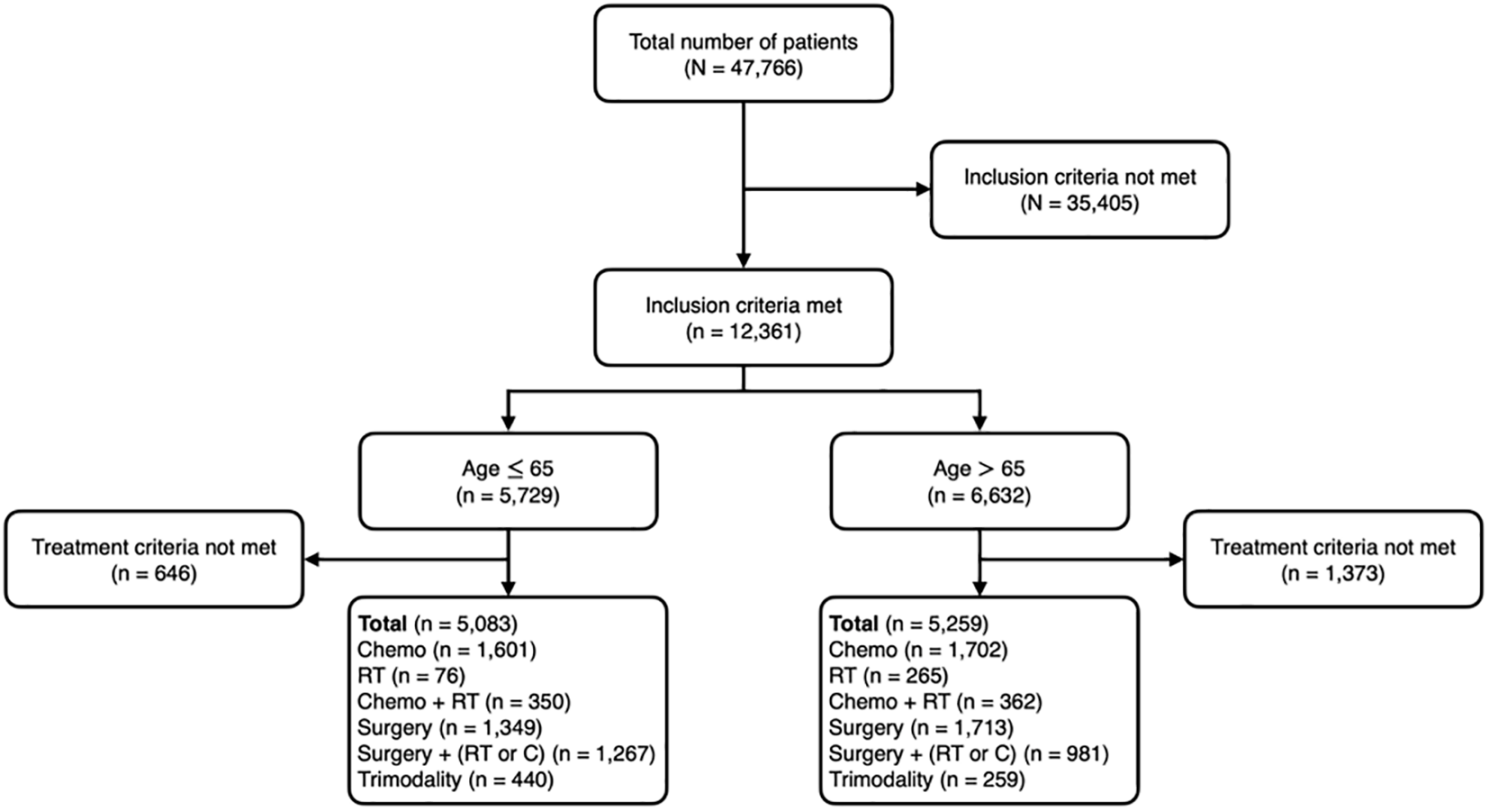
Flowchart for patient selection based on inclusion criteria and treatment modality. Chemo, chemotherapy, RT, Radiation therapy.

This study identified patients diagnosed with ICC from 2004 to 2020 based on the ICD-O-3 (International Classification of Diseases for Oncology, Third Edition) topography code C221, which corresponds to malignancy in the intrahepatic bile duct. Patients were subsequently filtered according to the inclusion and exclusion criteria summarized in **Figure 1**. Patients were included if they had known non-metastatic disease (clinical stage < IV), had available vital statistics data, and survived at least four months from the date of initial diagnosis. Patients were then stratified into two groups based on age > 65 years and age ≤ 65 years. Patients were then analyzed in treatment groups based on the combination of modalities received, as recorded in the NCDB. Treatment groups consisted of surgery only, chemotherapy only, radiotherapy (RT) only, chemotherapy plus RT, surgery plus chemotherapy or RT, and trimodality (surgery plus chemotherapy plus RT). RT was only considered as a primary treatment modality in this analysis if the total dose received by the patient was greater than 20 Gy, to exclude most palliative treatments. Patients who did not meet criteria for any of the pre-defined treatment groups above were excluded from the subsequent analyses.

### Statistical analysis

Kaplan-Meier (KM) survival curves were estimated for each treatment group and plotted with right-censored data. 95% confidence intervals (CI) for the instantaneous survival probability were calculated at each time point. Differences in overall survival across the six treatment groups were first assessed using a global multivariate log-rank test. Pairwise comparisons were subsequently conducted, with p-values adjusted for multiple comparisons using the Holm-Bonferroni method applied per time point considered (12 months, 24 months, and 60 months). The proportional hazards assumption was assessed for each treatment group within an age stratum by evaluating the correlation between scaled Schoenfeld residuals and ranked survival time, where a statistically significant correlation (p < 0.05) indicates a violation of the assumption. The proportional hazards assumption was valid within the surgical (surgery only, surgery + (chemo or RT), and trimodality) and the non-surgical (chemotherapy only, chemotherapy plus RT, RT only) treatment groups. Proportional hazards could not be assumed between surgical and non-surgical groups so their hazard ratios (HRs) could not be reliably calculated. HRs were estimated using Cox proportional hazards regression with surgery alone as the reference group for surgical treatments and chemotherapy alone for non-surgical treatments. Surgery alone and chemotherapy were selected as the reference groups because they each represent the most standard treatment approach for resectable and non-resectable treatments, respectively. Surgery alone and chemotherapy alone also had the largest sample sizes among their respective treatment subgroups, thus maximizing the precision of comparisons. To evaluate the potential influence of age on effect modification, HRs were estimated separately for older and younger patient subgroups. A p-value for interaction (p-interaction) was calculated to formally test whether age subgroup had a statistically significant influence on the underlying treatment effect. Statistical significance was defined as p < 0.10 for p-interaction given the generally low power for p-interaction analyses and p < 0.05 for all other tests. All reported p-values are two-sided. All statistical analyses were performed using Python (version 3.13) with the lifelines (version 0.30) (5) and statsmodels (version 0.14) (6) packages.

## Results

In total, 10,342 patients were identified and included in this study, with 5,259 (50.9%) stratified into an elderly (age > 65 years) cohort, and 5,038 (49.1%) stratified into a young (age ≤ 65 years) cohort (**Figure 1**). Among patients with operable disease, treatment patterns differed significantly between age groups (χ² p < 0.001), with elderly patients less likely to receive additional therapy with either trimodality therapy (8.8% vs 14.4%) or bimodality therapy (33.2% vs 41.5%) and more likely to undergo surgery alone (58.0% vs 44.1%). Among patients with inoperable disease, treatment patterns also differed significantly by age (χ² p < 0.001). Elderly patients were more likely to receive radiation alone (7.2% vs 2.8%) and less likely to receive chemotherapy-based approaches, including chemoradiation (9.8% vs 13.1%) and chemotherapy only (46.3% vs 59.9%) (**Table 1**).

**Table 1:** Treatment patterns and 1-, 2-, and 5-year overall survival outcomes by age and treatment modality.

Treatment group	Young (< 65 years)				Elderly (≥ 65 years)			
	n (%)	1-yr OS (95% CI)	2-yr OS (95% CI)	5-yr OS (95% CI)	n (%)	1-yr OS (95% CI)	2-yr OS (95% CI)	5-yr OS (95% CI)
• Surgical Treatments								
Trimodality	440 (14%)	93% (90–95%)	73% (69–77%)	43% (37–48%)	259 (9%)	91% (86–94%)	70% (64–75%)	45% (38–52%)
Surgery + (RT or Chemo)	1,267 (41%)	90% (88–91%)	73% (70–75%)	42% (38–45%)	981 (33%)	89% (87–91%)	72% (69–75%)	40% (36–44%)
Surgery alone	1,349 (44%)	89% (88–91%)	75% (73–78%)	49% (46–52%)	1,713 (58%)	88% (86–89%)	72% (70–74%)	41% (39–44%)
All surgical patients	3,056 (53%)				2,953 (45%)			
• Non-surgical Treatments								
Chemo only	1,601 (60%)	64% (62–67%)	34% (32–37%)	11% (9–13%)	1,702 (46%)	58% (56–61%)	26% (24–28%)	5% (4–7%)
RT only	76 (3%)	58% (46–69%)	41% (29–52%)	18% (9–29%)	265 (7%)	67% (60–72%)	45% (38–51%)	11% (7–16%)
Chemo + RT	350 (13%)	70% (65–75%)	41% (36–47%)	15% (11–20%)	362 (10%)	68% (63–72%)	37% (32–42%)	8% (5–13%)
Other	646 (24%)				1,350 (37%)			
All non-surgical patients	2,673 (47%)				3,679 (55%)			
All patients	5,729 (100%)				6,632 (100%)			

Treatment-stratified baseline characteristics demonstrated important imbalances across treatment groups. Among surgically managed patients (**Table 2**), those receiving trimodality therapy had higher-risk disease features compared with those undergoing surgery alone. In both age cohorts, trimodality therapy was associated with a higher proportion of AJCC stage III disease and positive surgical margins. Among young patients, stage III disease was present in 33.2% of trimodality patients compared with 14.0% of surgery-only patients, and positive margins were present in 41.4% versus 10.0%, respectively. Among elderly patients, stage III disease was present in 36.3% of trimodality patients compared with 14.1% of surgery-only patients, and positive margins were present in 45.2% versus 10.2%, respectively. These findings suggest that patients selected for treatment escalation after surgery had more adverse baseline clinicopathologic features. Additional baseline characteristics in patients treated with non-surgical approaches are shown in **Table 3**.

**Table 2:** Clinicodemographic and disease characteristics of patients who underwent surgery.

Treatment		Trimodality		Surg + (Chemo/RT)		Surgery Only	
Age group		Young (≤65)	Elderly (>65)	Young (≤65)	Elderly (>65)	Young (≤65)	Elderly (>65)
Total N		440 (100.0%)	259 (100.0%)	1267 (100.0%)	981 (100.0%)	1349 (100.0%)	1713 (100.0%)
Sex	Male	229 (52.0%)	137 (52.9%)	586 (46.3%)	454 (46.3%)	671 (49.7%)	822 (48.0%)
	Female	211 (48.0%)	122 (47.1%)	681 (53.7%)	527 (53.7%)	678 (50.3%)	891 (52.0%)
Race	White	374 (85.0%)	231 (89.2%)	1041 (82.2%)	858 (87.5%)	1097 (81.3%)	1477 (86.2%)
	Black	29 (6.6%)	7 (2.7%)	96 (7.6%)	48 (4.9%)	135 (10.0%)	97 (5.7%)
	Other	31 (7.0%)	20 (7.7%)	123 (9.7%)	67 (6.8%)	105 (7.8%)	120 (7.0%)
	Unknown	6 (1.4%)	1 (0.4%)	7 (0.6%)	8 (0.8%)	12 (0.9%)	19 (1.1%)
Hispanic Origin	Non-Hispanic	394 (89.5%)	244 (94.2%)	1144 (90.3%)	877 (89.4%)	1217 (90.2%)	1557 (90.9%)
	Hispanic	38 (8.6%)	8 (3.1%)	107 (8.4%)	84 (8.6%)	97 (7.2%)	100 (5.8%)
	Unknown	8 (1.8%)	7 (2.7%)	16 (1.3%)	20 (2.0%)	35 (2.6%)	56 (3.3%)
Insurance Status	Not insured	11 (2.5%)	0 (0.0%)	30 (2.4%)	1 (0.1%)	42 (3.1%)	6 (0.4%)
	Private	341 (77.5%)	32 (12.4%)	869 (68.6%)	122 (12.4%)	791 (58.6%)	183 (10.7%)
	Medicaid	27 (6.1%)	5 (1.9%)	129 (10.2%)	13 (1.3%)	161 (11.9%)	22 (1.3%)
	Medicare	41 (9.3%)	218 (84.2%)	191 (15.1%)	828 (84.4%)	278 (20.6%)	1449 (84.6%)
	Other govt	10 (2.3%)	2 (0.8%)	28 (2.2%)	12 (1.2%)	36 (2.7%)	17 (1.0%)
	Unknown	10 (2.3%)	2 (0.8%)	20 (1.6%)	5 (0.5%)	41 (3.0%)	36 (2.1%)
Income	Quartile 1 (low)	61 (13.9%)	36 (13.9%)	172 (13.6%)	97 (9.9%)	200 (14.8%)	222 (13.0%)
	Quartile 2	73 (16.6%)	44 (17.0%)	181 (14.3%)	162 (16.5%)	263 (19.5%)	278 (16.2%)
	Quartile 3	83 (18.9%)	54 (20.8%)	243 (19.2%)	204 (20.8%)	248 (18.4%)	399 (23.3%)
	Quartile 4 (high)	162 (36.8%)	84 (32.4%)	475 (37.5%)	368 (37.5%)	451 (33.4%)	584 (34.1%)
	Unknown	61 (13.9%)	41 (15.8%)	196 (15.5%)	150 (15.3%)	187 (13.9%)	230 (13.4%)
CD Score	Score 0	326 (74.1%)	180 (69.5%)	857 (67.6%)	614 (62.6%)	859 (63.7%)	996 (58.1%)
	Score 1	71 (16.1%)	54 (20.8%)	266 (21.0%)	215 (21.9%)	294 (21.8%)	417 (24.3%)
	Score 2	25 (5.7%)	17 (6.6%)	72 (5.7%)	79 (8.1%)	92 (6.8%)	158 (9.2%)
	Score 3+	18 (4.1%)	8 (3.1%)	72 (5.7%)	73 (7.4%)	104 (7.7%)	142 (8.3%)
AJCC Stage	Stage 0	3 (0.7%)	0 (0.0%)	10 (0.8%)	0 (0.0%)	2 (0.1%)	3 (0.2%)
	Stage I	166 (37.7%)	83 (32.0%)	446 (35.2%)	413 (42.1%)	786 (58.3%)	1011 (59.0%)
	Stage II	125 (28.4%)	82 (31.7%)	486 (38.4%)	315 (32.1%)	372 (27.6%)	458 (26.7%)
	Stage III	146 (33.2%)	94 (36.3%)	325 (25.7%)	253 (25.8%)	189 (14.0%)	241 (14.1%)
Surgical Margin	R0	237 (53.9%)	122 (47.1%)	909 (71.7%)	675 (68.8%)	1076 (79.8%)	1303 (76.1%)
	≥ R1	182 (41.4%)	117 (45.2%)	231 (18.2%)	201 (20.5%)	135 (10.0%)	175 (10.2%)
	No surgery	0 (0.0%)	0 (0.0%)	0 (0.0%)	0 (0.0%)	0 (0.0%)	0 (0.0%)
	Unknown	21 (4.8%)	20 (7.7%)	127 (10.0%)	105 (10.7%)	138 (10.2%)	235 (13.7%)
Surgical status	Surgery performed	440 (100.0%)	259 (100.0%)	1267 (100.0%)	981 (100.0%)	1349 (100.0%)	1713 (100.0%)
	Not recommended	0 (0.0%)	0 (0.0%)	0 (0.0%)	0 (0.0%)	0 (0.0%)	0 (0.0%)
	Contraindicated	0 (0.0%)	0 (0.0%)	0 (0.0%)	0 (0.0%)	0 (0.0%)	0 (0.0%)
	Other/Unknown	0 (0.0%)	0 (0.0%)	0 (0.0%)	0 (0.0%)	0 (0.0%)	0 (0.0%)

**Table 3:** Clinicodemographic and disease characteristics of patients treated with nonsurgical approaches.

Treatment		Chemo + RT		Chemo only		RT only	
Age group		Young (≤65)	Elderly (>65)	Young (≤65)	Elderly (>65)	Young (≤65)	Elderly (>65)
Total N		350 (100.0%)	362 (100.0%)	1601 (100.0%)	1702 (100.0%)	76 (100.0%)	265 (100.0%)
Sex	Male	195 (55.7%)	174 (48.1%)	792 (49.5%)	817 (48.0%)	45 (59.2%)	122 (46.0%)
	Female	155 (44.3%)	188 (51.9%)	809 (50.5%)	885 (52.0%)	31 (40.8%)	143 (54.0%)
Race	White	288 (82.3%)	314 (86.7%)	1299 (81.1%)	1484 (87.2%)	62 (81.6%)	234 (88.3%)
	Black	30 (8.6%)	23 (6.4%)	190 (11.9%)	110 (6.5%)	8 (10.5%)	13 (4.9%)
	Other	29 (8.3%)	24 (6.6%)	95 (5.9%)	93 (5.5%)	5 (6.6%)	17 (6.4%)
	Unknown	3 (0.9%)	1 (0.3%)	17 (1.1%)	15 (0.9%)	1 (1.3%)	1 (0.4%)
Hispanic Origin	Non-Hispanic	308 (88.0%)	323 (89.2%)	1410 (88.1%)	1536 (90.2%)	63 (82.9%)	236 (89.1%)
	Hispanic	29 (8.3%)	21 (5.8%)	138 (8.6%)	116 (6.8%)	10 (13.2%)	14 (5.3%)
	Unknown	13 (3.7%)	18 (5.0%)	53 (3.3%)	50 (2.9%)	3 (3.9%)	15 (5.7%)
Insurance Status	Not insured	10 (2.9%)	5 (1.4%)	56 (3.5%)	10 (0.6%)	1 (1.3%)	1 (0.4%)
	Private	230 (65.7%)	33 (9.1%)	972 (60.7%)	176 (10.3%)	35 (46.1%)	26 (9.8%)
	Medicaid	34 (9.7%)	6 (1.7%)	203 (12.7%)	32 (1.9%)	9 (11.8%)	3 (1.1%)
	Medicare	53 (15.1%)	308 (85.1%)	289 (18.1%)	1437 (84.4%)	27 (35.5%)	222 (83.8%)
	Other govt	12 (3.4%)	9 (2.5%)	24 (1.5%)	18 (1.1%)	3 (3.9%)	7 (2.6%)
	Unknown	11 (3.1%)	1 (0.3%)	57 (3.6%)	29 (1.7%)	1 (1.3%)	6 (2.3%)
Income	Quartile 1 (low)	50 (14.3%)	46 (12.7%)	241 (15.1%)	211 (12.4%)	8 (10.5%)	39 (14.7%)
	Quartile 2	66 (18.9%)	53 (14.6%)	288 (18.0%)	309 (18.2%)	14 (18.4%)	44 (16.6%)
	Quartile 3	58 (16.6%)	88 (24.3%)	342 (21.4%)	339 (19.9%)	19 (25.0%)	49 (18.5%)
	Quartile 4 (high)	115 (32.9%)	126 (34.8%)	503 (31.4%)	612 (36.0%)	21 (27.6%)	83 (31.3%)
	Unknown	61 (17.4%)	49 (13.5%)	227 (14.2%)	231 (13.6%)	14 (18.4%)	50 (18.9%)
CD Score	Score 0	265 (75.7%)	241 (66.6%)	1160 (72.5%)	1120 (65.8%)	47 (61.8%)	149 (56.2%)
	Score 1	58 (16.6%)	76 (21.0%)	294 (18.4%)	366 (21.5%)	14 (18.4%)	52 (19.6%)
	Score 2	17 (4.9%)	25 (6.9%)	67 (4.2%)	124 (7.3%)	6 (7.9%)	25 (9.4%)
	Score 3+	10 (2.9%)	20 (5.5%)	80 (5.0%)	92 (5.4%)	9 (11.8%)	39 (14.7%)
AJCC Stage	Stage 0	1 (0.3%)	0 (0.0%)	3 (0.2%)	4 (0.2%)	0 (0.0%)	0 (0.0%)
	Stage I	96 (27.4%)	112 (30.9%)	314 (19.6%)	427 (25.1%)	39 (51.3%)	142 (53.6%)
	Stage II	97 (27.7%)	105 (29.0%)	617 (38.5%)	715 (42.0%)	21 (27.6%)	83 (31.3%)
	Stage III	156 (44.6%)	145 (40.1%)	667 (41.7%)	556 (32.7%)	16 (21.1%)	40 (15.1%)

**Figure 2:**
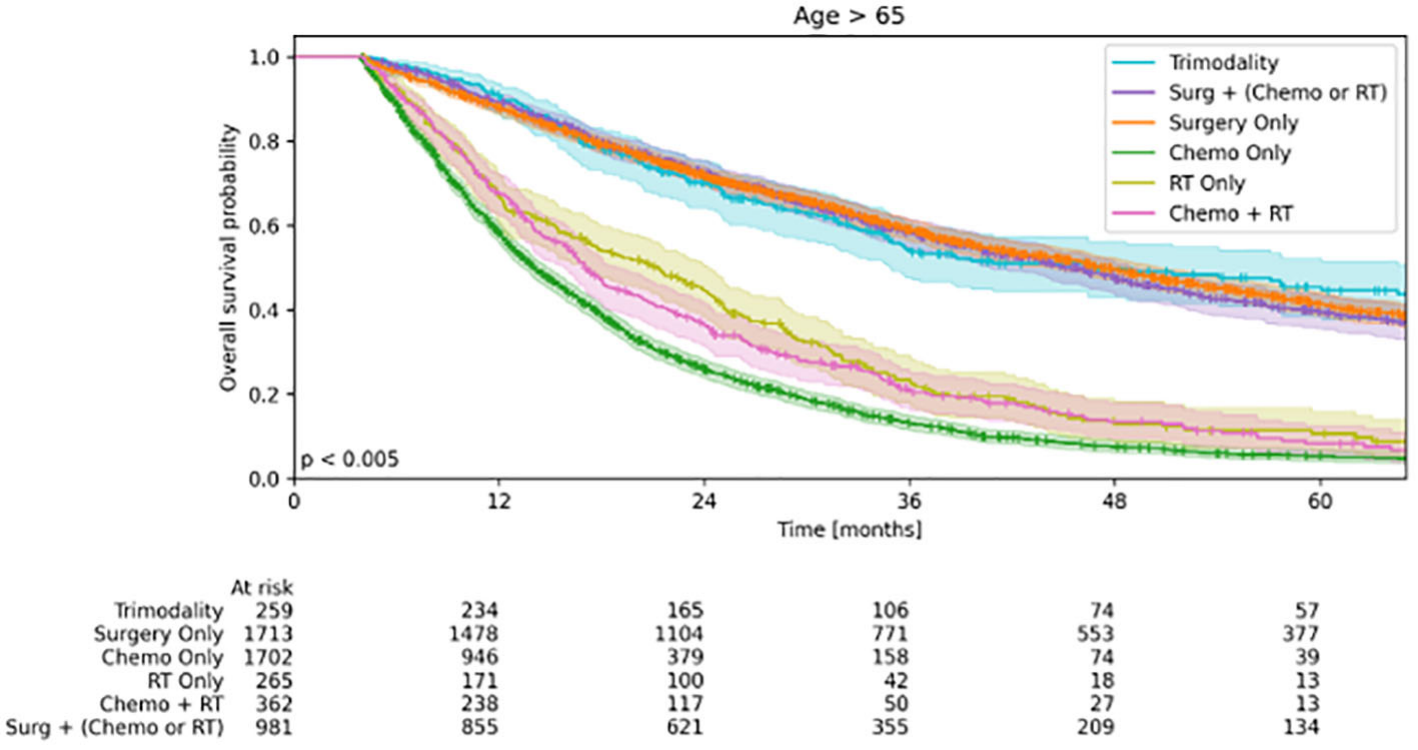
Kaplan Meier curve of elderly patients stratified by treatment modality.

**Figure 3:**
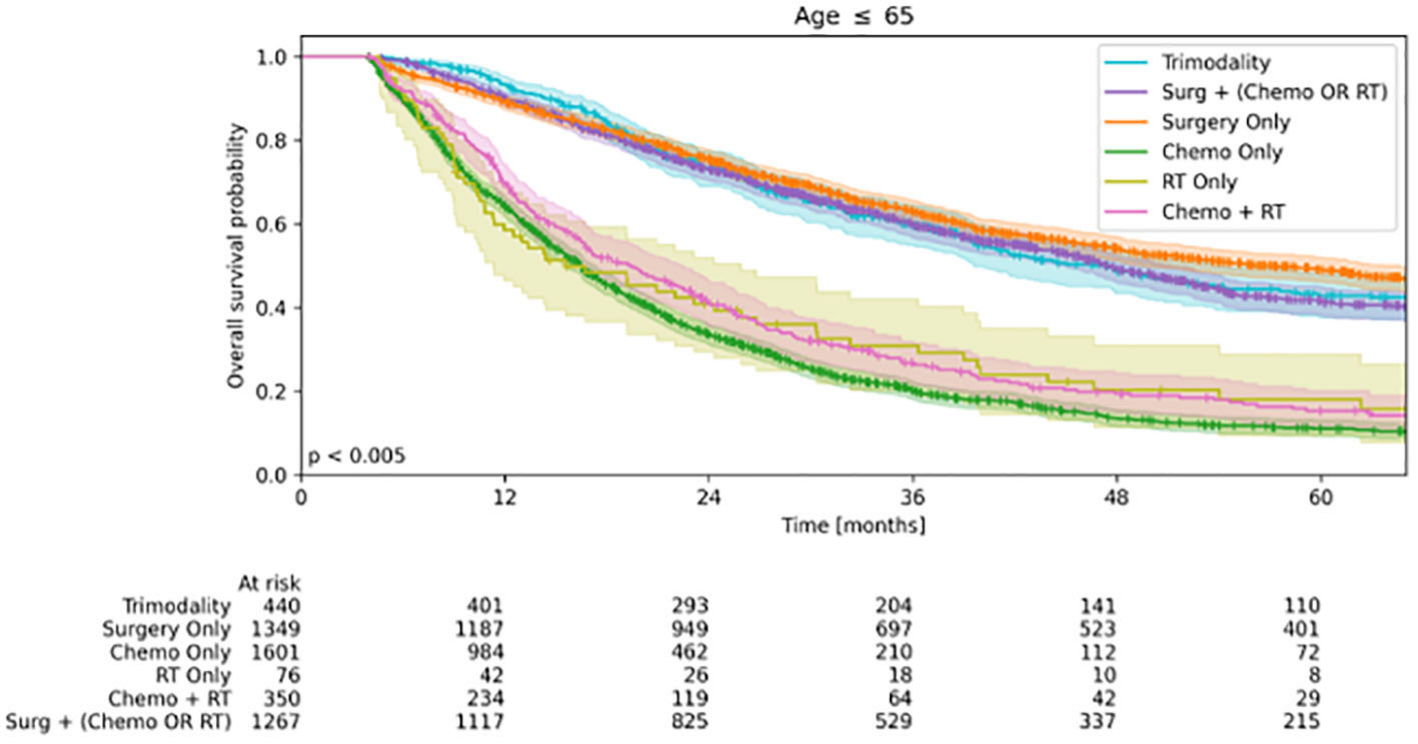
Kaplan Meier curve of younger patients stratified by treatment modality.

Within each age cohort, there was at least one significant difference between the Kaplan-Meier survival curves of the six treatment modalities considered, which prompted multiple comparisons testing for 1-year, 2-year, and 5-year overall survival (OS) (**Figures 2**, **3**). In both elderly and young cohorts, all treatment interventions that involved primary surgical resection (i.e., surgery only, surgery and chemotherapy or RT, trimodality) resulted in significant overall survival benefit at all time points considered compared to non-surgical treatments. In the elderly cohort, there was no significant difference in 1-year, 2-year, and 5-year OS between the three surgical interventions (surgery only, surgery + (chemo or RT), trimodality). The results are similar for 1-year and 2-year OS in the young cohort, but there was a significant increase in 5-year OS for the surgery only group at 49.1% (95% CI 46.1–52.0%) compared to 41.6% (95% CI 38.2–45.0%, p=0.006) in the Surgery + (Chemo or RT) group. There was a trend but no statistically significant difference in 5-year OS in the surgery only group compared to 42.8% (95% CI 37.5–48.0%, p=0.200) in the trimodality group.

Between treatment interventions that did not involve a primary surgical resection (i.e., chemo only, RT only, chemo + RT), it is notable that in the elderly cohort, the 1-year OS and 2-year OS in the chemo + RT group were significantly higher compared to that of the chemo only group, whereas only the 2-year OS in the RT only group was significantly higher than that of the chemo only group. The 1-year and 2-year OS was 67.6% (95% CI 62.5–72.1%, p=0.009) and 36.4% (95% CI 31.3–41.5%, p=0.001) for the chemo + RT group compared to 58.3% (95% CI 55.9–60.6%) and 26.0% (95% CI 23.9–28.2%) for the chemo only group, respectively. Interestingly, the RT only group demonstrated improved survival outcomes compared to the chemo only group and comparable to the chemo + RT group. There was no significant difference in 5-year OS between non-surgical interventions in the elderly cohort. In the younger cohort, all nonsurgical interventions had equivocal outcomes for 1-year, 2-year, and 5-year OS.

**Figure 4:**
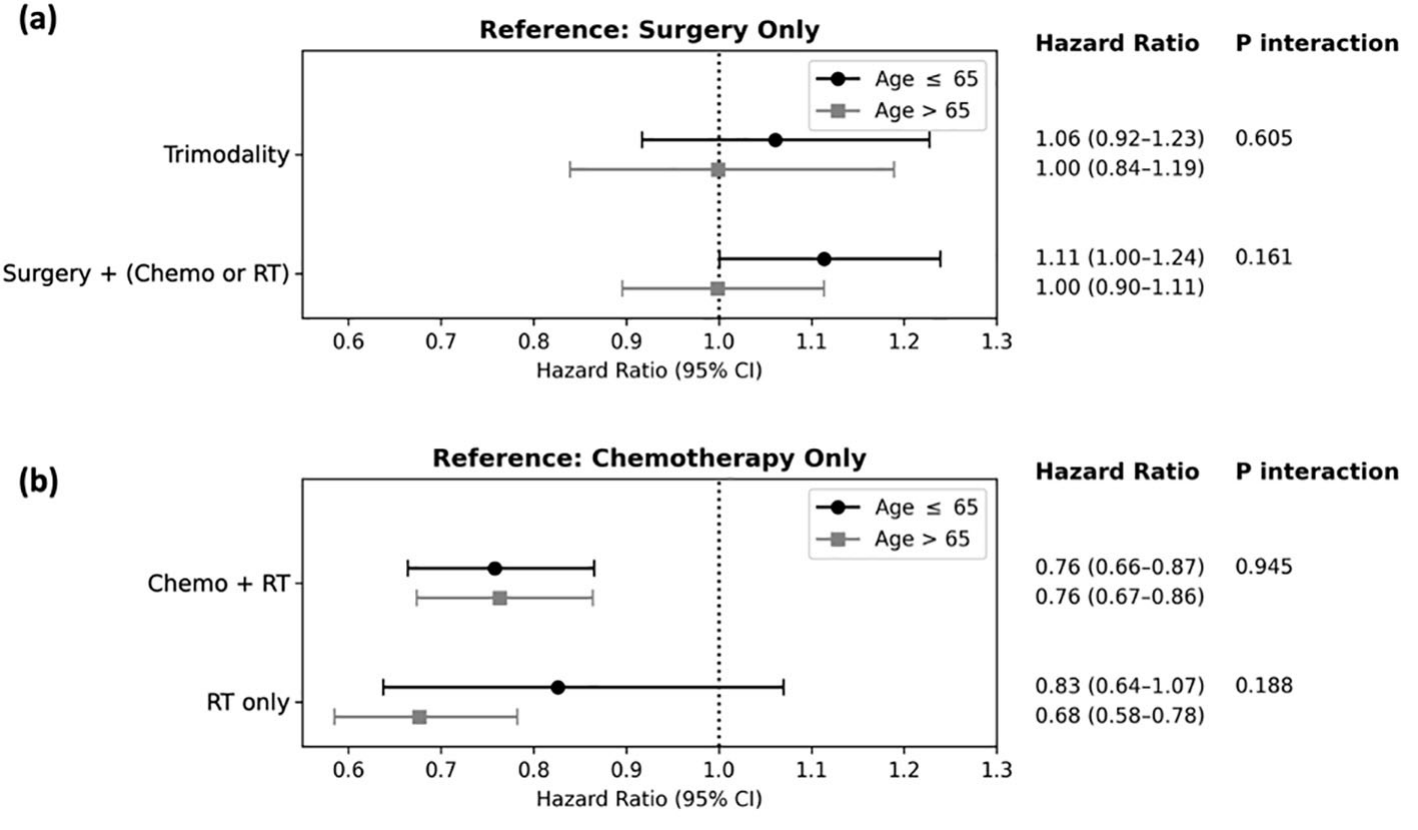
Forest plot, hazard ratios, and p interaction by treatment modality, stratified by age group. **(a)** Comparison of trimodality and surgery plus chemotherapy or RT, referenced to surgery only. **(b)** Comparison of RT only and chemotherapy plus RT, referenced to chemotherapy only.

The hazard ratios (HR) for OS of the treatment modalities stratified by age group are shown in **Figure 4**. Compared to surgery only (**Figure 4a**), the HR for OS of elderly patients in the surgery + (chemo or RT) group was 1.00 (95% CI 0.90–1.11) and the HR of the trimodality group was 1.00 (95% CI 0.84–1.19). In comparison, the analogous hazard ratio (HR) for OS of young patients in the surgery + (chemo or RT) group was 1.11 (95% CI 1.00–1.24) while that of the trimodality group was 1.06 (95% CI 0.92–1.23). Compared to chemotherapy only (**Figure 4b**), the HR for OS of elderly patients in the chemo + RT group was 0.76 (95% CI 0.67–0.86) and the HR of the RT only group was 0.68 (95% CI 0.58–0.78). In comparison, the analogous hazard ratio (HR) for OS of young patients in the chemo + RT group was 0.76 (95% CI 0.66–0.87) while that of the RT only group was 0.83 (95% CI 0.64–1.07). There was no significant effect from age stratification to the HRs of a given intervention since all P interaction terms > 0.10 when we consider a higher significance threshold in a low-powered interaction analysis.

## Discussion

In this retrospective NCDB analysis of patients with nonmetastatic intrahepatic cholangiocarcinoma, we observed statistically significant age-associated differences in treatment patterns, with elderly patients receiving less intensive therapy across both surgical and nonsurgical settings. Among patients who underwent surgery, elderly patients were less likely to receive multimodality therapy and more likely to undergo surgery alone. Similarly, among patients managed without surgery, elderly patients were less likely to receive chemotherapy-based treatment and more likely to receive radiation monotherapy. Collectively, these findings demonstrated an association between older age and treatment de-intensification, particularly with respect to systemic therapy use. However, given the observational nature of this study, these patterns should not be interpreted as evidence that de-intensified therapy is equivalent or preferable in elderly patients, but rather as a reflection of real-world treatment selection in a population for whom prospective data remain limited.

Survival outcomes in this cohort were strongly associated with receipt of surgical resection. Across both age groups, treatment strategies incorporating surgery were associated with longer overall survival compared with nonsurgical approaches. However, this finding must be interpreted cautiously because surgical and nonsurgical patients likely represent clinically distinct populations. In particular, patients selected for surgery are more likely to have resectable disease, lower tumor burden, more favorable anatomy, better performance status, and fewer competing comorbidities, whereas patients managed without surgery may have unresectable disease or be medically inoperable. Therefore, the observed survival advantage among surgical patients should not be interpreted as a causal estimate of treatment efficacy relative to nonsurgical management, but rather as an expected association reflecting both treatment selection and underlying disease biology.

Among patients who underwent surgery, the addition of chemotherapy and/or radiotherapy was not associated with consistent improvements in survival relative to surgery alone. However, the treatment-stratified baseline characteristics demonstrate that patients receiving trimodality therapy had higher-risk disease features than patients treated with surgery alone, including higher AJCC stage and substantially higher rates of positive surgical margins. This pattern is consistent with confounding by indication, whereby treatment escalation was more frequently used for patients with adverse disease biology or higher-risk pathologic features. Therefore, the apparent similarity in survival between trimodality therapy and surgery alone may reflect multimodality therapy partially offsetting worse baseline prognostic factors, rather than an absence of therapeutic benefit.

Among patients managed without surgery, radiotherapy-based treatment was associated with favorable short-term survival outcomes in elderly patients. Specifically, chemoradiation was associated with improved 1-year and 2-year survival compared with chemotherapy alone, while radiation monotherapy demonstrated outcomes that appeared comparable to chemoradiation. Although these findings are clinically interesting, they should be interpreted as hypothesis-generating, as the scope of our study did not delve into specific associations of survival outcomes to patient and disease factors such as patient frailty, comorbidity burden, tumor extent, liver function, treatment intent, chemotherapy regimen, or sequencing of systemic therapy and radiotherapy. These unmeasured factors may have strongly influenced treatment selection and survival outcomes. Therefore, the observed association between radiotherapy-based approaches and survival in elderly nonsurgical patients should not be interpreted as evidence that radiotherapy alone is equivalent to chemoradiation, but rather as a signal that local therapy may merit further investigation in carefully selected elderly patients with unresectable ICC.

The findings of this study should be contextualized within the landmark trials defining current standard of care treatments for biliary tract cancers. The ABC I/II trials established gemcitabine plus cisplatin as the preferred option over gemcitabine alone for locally advanced or metastatic biliary tract cancers, demonstrating improvements in overall survival and progression free survival in per-protocol analysis (7, 8). However, the median age of the patients in the ABC I/II trial were younger, and there was no analysis on the relationship between patient age and treatment efficacy (8).

In the adjuvant setting, the BILCAP phase 3 trial demonstrated that capecitabine after R0 or R1 resection increased median overall survival (OS) by 14.7 months compared to observation. OS and relapse-free survival (RFS) benefits were demonstrated in the capecitabine group in a prespecified per-protocol analysis, but not in an ITT analysis (9). Similarly, the primary analysis of the ASCOT phase 3 trial demonstrated that adjuvant S-1 (oral fluoropyrimidine combination drug, not approved for use in the United States) after R0 or R1 resection provided a statistically significant benefit to survival versus observation in an ITT analysis of Asian patients (10). Importantly, neither study demonstrated significant heterogeneity in treatment effect based on age subgroups of patients aged ≤ 60 years and > 60 years, suggesting that elderly patients may derive similar relative benefit from adjuvant therapy as younger patients. On the contrary, our analysis did not demonstrate a consistent survival advantage with the addition of adjuvant therapy to surgery.

The role of trimodality therapy in biliary tract cancers remains less well defined. SWOG0809 was a single-arm phase 2 trial of surgically resected high-risk extrahepatic cholangiocarcinoma or gallbladder carcinoma treated with gemcitabine and capecitabine followed by chemoradiation. The 2-year OS was 65%, and the 2-year local relapse incidence was low at 11%. Both metrics exceeded historical expectations, and the treatment demonstrated tolerable toxicity (11). While this study shows promise for trimodality therapy for BTC, it notably did not include ICC. Nonetheless, it supports the broader concept that aggressive multimodality approaches may improve outcomes in well selected high-risk patients. In our analysis, trimodality therapy was not associated with improvement in survival, but these findings are likely limited by the lack of granularity of disease characteristics, patient and treatment selection, and outcome measures.

An important limitation of this analysis is the limited treatment granularity available within the NCDB. Although chemotherapy and radiotherapy receipt are captured, details regarding chemotherapy regimen, treatment intensity, sequencing, completion, and therapeutic intent are not reliably available. This is particularly relevant given the long study period from 2004 to 2020, during which systemic therapy standards for biliary tract cancers have evolved. Similarly, our definition of trimodality therapy as surgery plus chemotherapy plus radiotherapy encompasses heterogeneous treatment pathways, including neoadjuvant, adjuvant, sequential, and concurrent approaches that may carry different prognostic implications. Therefore, outcomes associated with chemotherapy-containing and trimodality approaches should be interpreted as broad associations with treatment receipt rather than evidence for any specific regimen, sequence, or treatment strategy.

More broadly, a recent review on BTC treatment in elderly patients indicates that while there is limited data on age-specific treatment outcomes, available clinical trial data do not indicate any significant difference in survival outcomes based on age (12). Despite this, elderly patients in our cohort were consistently less likely to receive escalated therapy, a pattern likely reflecting a combination of factors, including increased comorbidity burden, concerns regarding treatment tolerance, and the absence of robust prospective data to guide decision-making in this population. Both NCCN and ASCO guidelines recommend that elderly patients be given a formal geriatric assessment of function to help clinical decision-making around escalating care in oncological disease management (13). Greater incorporation of these assessments into routine clinical practice may improve patient selection for multimodality therapy and help ensure that treatment decisions are guided by physiologic rather than chronologic age.

Taken together, these findings suggest that while surgery remains strongly associated with survival in patients with resectable ICC, elderly patients are less likely to receive multimodality or systemic therapy, even in settings where such treatments may be beneficial. In the inoperable setting, radiation therapy may serve a crucial role in achieving local control and improving short-term survival outcomes. These results underscore the need for more prospective studies to better define optimal treatment strategies for elderly patients with ICC and support a more individualized approach to treatment selection that incorporates both oncologic and patient-specific factors.

## Data Availability

The raw data supporting the conclusions of this article will be made available by the authors, without undue reservation.
